# Standardizing metadata and taxonomic identification in metabarcoding studies

**DOI:** 10.1186/s13742-015-0074-5

**Published:** 2015-07-31

**Authors:** Leho Tedersoo, Kelly S. Ramirez, R Henrik Nilsson, Aivi Kaljuvee, Urmas Kõljalg, Kessy Abarenkov

**Affiliations:** 1 Natural History Museum, University of Tartu, 14a Ravila, 50411 Tartu, Estonia; 2 Netherlands Institute of Ecology, Droevendaalsesteeg 10, 6708 PB Wageningen, The Netherlands; 3 Department of Biological and Environmental Sciences, University of Gothenburg, Box 461, 405 30 Gothenburg, Sweden; 4 Institute of Ecology and Earth Sciences, University of Tartu, 14a Ravila, 50411 Tartu, Estonia

**Keywords:** High-throughput sequencing (HTS), Next-generation sequencing, Data storage, Environmental metadata, Species hypotheses, Digital object identifiers (DOI), Internal transcribed spacer (ITS), Interactive database

## Abstract

High-throughput sequencing-based metabarcoding studies produce vast amounts of ecological data, but a lack of consensus on standardization of metadata and how to refer to the species recovered severely hampers reanalysis and comparisons among studies. Here we propose an automated workflow covering data submission, compression, storage and public access to allow easy data retrieval and inter-study communication. Such standardized and readily accessible datasets facilitate data management, taxonomic comparisons and compilation of global metastudies.

## Background

The revolution of high-throughput sequencing (HTS) technologies has provided an unprecedented insight into the biodiversity and ecology of organisms, particularly those that are microscopic and difficult to culture. Such metabarcoding studies from terrestrial and aquatic ecosystems, as well as from living organisms, generate enormous amounts of sequence data and associated environmental metadata to address the properties of biodiversity. However, comparisons among datasets and global syntheses are severely hampered by the widespread use of different protocols in virtually every step of the data generation and analysis processes, from sample preparation through laboratory work to calculation of operational taxonomic units (OTUs), the proxies for species [1]. The naming of these OTUs is non-systematic and study-specific, which disables wide-scale taxonomic communication [2]. Furthermore, the outcome of the calculation of OTUs is strongly dependent on the choice of bioinformatics tools and clustering methods. Improved bioinformatics techniques reveal systematic errors in HTS datasets, and their reanalysis opens new perspectives in understanding biodiversity [3, 4]. The possibility of recalculation and integration of data into metastudies is of great importance to understand the patterns of biodiversity over large spatiotemporal scales [5].

Compilation of metastudies requires easy access to environmental metadata and sequence data. Yet the metadata describing sampling location, habitat quality and interacting taxa are often excluded from the published supplementary datasets. Alternatively, these data remain buried in inaccessible forms in databases such as the Short Read Archive (SRA) [www.ncbi.nlm.nih.gov/sra], DataDryad [http://datadryad.org], or in spreadsheets on the authors’ computers [4]. For example, of 27 fungal metabarcoding datasets we tried to access, many were missing from public repositories (37 %), were locked (19 %), or lacked information about de-multiplexing samples (15 %). Contacting the corresponding authors finally enabled us to recover 67 % of the requested data. Furthermore, even if the environmental metadata are available, they are typically located in user-defined data fields with different units or no units at all.

**Fig. 1:**
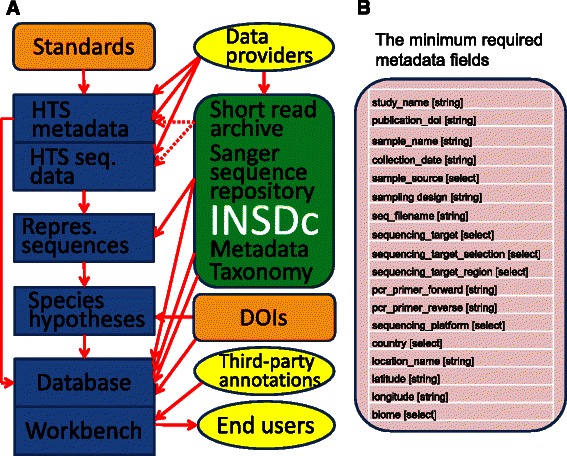
General data structure. **a** Suggested workflow using various bioinformatics tools and databases. DOI, digital object identifier; HTS, high-throughput sequencing; INSDc, International Nucleotide Sequence Database Collaboration; repres, representative; seq, sequencing. **b** Proposed minimum data fields for HTS metadata

Building on these shortcomings, existing standards, and recently developed bioinformatics tools, we propose a workflow for standardized metadata and sequence data synthesis (Fig. 1). This workflow has the potential to form a basis for efficient data management, download, and scientific hypothesis testing in taxonomic and ecological metastudies.

### Data standardization

Long-established disciplines such as plant and animal sciences have a rich history of standardization, with Darwin Core [6] and the Access to Biological Collections Data (ABCD) [www.tdwg.org] representing the most widely used formats. These efforts have demonstrated the benefit of data standardization, particularly the ability to address research and management questions relevant to global change [5]. Initiatives such as the International Nucleotide Sequence Database Collaboration (INSDC) [www.insdc.org], Global Biodiversity Information Facility (GBIF) [www.gbif.org], Map of Life [www.mapoflife.org], Encyclopedia of Life [www.eol.org], International Barcode of Life (iBOL) [http://ibol.org], and Data Observation Network for Earth (DataONE) [www.dataone.org] constitute the global leaders in managing biodiversity information. Conversely, microbial ecology and other disciplines using metabarcoding tools for species identification have had no organized standardization efforts until recently. ‘Minimum information about a marker gene sequence’ (MIMARKS) is a recent effort developed by the Genomics Standards Consortium to implement standardization and description of sequence-based data [7].

Still, agreement on global standards does not automatically ensure enforcement. Providing data with scientific publications has long been encouraged, but leading publishers have only recently declared this a strict requirement. Unfortunately, this effort has not extended to standardization. Therefore, we advocate that journals and institutes should follow a set of standards agreed upon by the research community [1]. We recommend that metadata terminology should follow the MIMARKS and Darwin Core standards, because these formats have been generally approved and implemented by multiple initiatives and thus provide compatibility. Therefore, the database modules for storing sequence data and metadata need to be very broad and flexible to cover virtually all fields of biology. Similarly, the forms for data upload should be simple and follow both the above standards. To simplify upload of sequence data and metadata, we propose a single spreadsheet for metainformation about the whole study (studies), site(s) and sample(s) as well as links to demultiplexed HTS files (Fig. 1).

Efficient data management requires an interactive database associated with an online workbench for data curation, annotation and analysis. Because of its central role in data storage and well-developed modules for up-to-date taxonomy, the INSDC would preferably lead this development. Multiple commitments and paucity of directed funding mean that the INSDC evolves slowly and thus remains badly behind the research needs. Therefore, research consortia have generated multiple platforms for data storage, bioinformatics analysis and statistical analysis, e.g. Quantitative Insights into Microbial Ecology (QIIME) [www.qiime.org], Ribosomal Database Project (RDP) [https://rdp.cme.msu.edu/], Silva [www.arb-silva.de/], Barcode of Life Data Systems (BOLD Systems) [www.boldsystems.org], and UNITE [https://unite.ut.ee]. The two latter platforms are suitably structured for managing barcoding data. Unfortunately, these initiatives remain largely unconnected. Integrating these community-driven efforts to create a central data portal requires interdisciplinary collaboration involving expert knowledge from multiple research fields to bridge the disciplines of taxonomy and ecology of organisms with genomics and bioinformatics.

### Communication of species

Raw HTS data analysis is time consuming and requires substantial bioinformatics skills. To undertake comparisons across metabarcoding studies, noise removal and OTU calculation must be done following the same bioinformatics protocols [8]. This effectively disables comparisons across HTS platforms, which differ in base calling and error rates. Thus, if all HTS sequence authors were to reduce the size and complexity of their data before adding them to repositories, those data would be much more useful to the wider research community. The processing of uploaded data generated by different HTS technologies needs the implementation of standardized automated bioinformatics routines. The research community requires a consensus on how to implement these bioinformatics tools in a way that finds the middle ground among minimizing technical errors and tag switches, maximizing the sensitivity to biological variation, and downstream reanalysis requirements. Given that most HTS platforms produce sequences with error rates approaching 1 %, we propose that sequence data be clustered at 99 % similarity using single linkage methods. Representative sequences of non-singleton taxonomic clusters should be selected automatically, based on their similarity to the consensus sequence of the cluster and should carry information about relative abundance. Researchers wishing to use other similarity thresholds and algorithms can rapidly process these deposited representative sequences to that effect. The continuous development of bioinformatics HTS data analysis tools means that it is also necessary to store raw data to enable future recalculation of clusters and representative sequences.

**Fig. 2:**
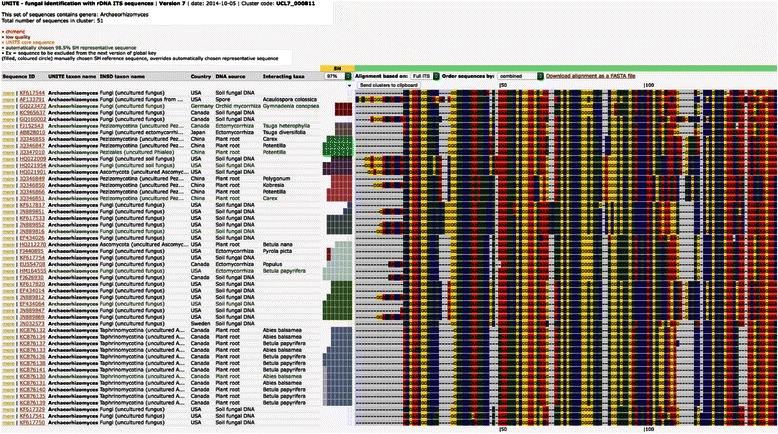
Screenshot of PlutoF workbench [11] for managing species hypotheses in the UNITE database [https://unite.ut.ee]. Multiple alignment of one of 20 clades of the enigmatic fungal class Archaeorhizomycetes is shown. Species hypotheses (SH) based on 97.0-100.0 % sequence similarity thresholds are marked with color patterns. The representative sequence of each SH is shown in green text. User-annotated taxonomic and ecological metadata are also indicated

To provide centralized species identification, representative sequences of metabarcoding studies should be further clustered along with Sanger sequences using multiple sequence similarity thresholds that represent species hypotheses [9]. This approach allows users to choose the biologically meaningful OTUs for further downstream analyses (Fig. 2). For direct taxonomic communication of OTUs across studies, we recommend the use of digital object identifiers (DOI) of species hypotheses. Such a system was recently introduced in the UNITE platform for Sanger sequences of the internal transcribed spacer (ITS) region, the official fungal barcode [9]. Several HTS bioinformatics workbenches such as QIIME and mothur [www.mothur.org] have implemented the use of species hypotheses for fungal ITS sequences. Similarly, the BOLD system has implemented fixed-threshold species approximation and the barcode index numbers (BIN) naming system for the Cytochrome C Oxidase subunit 1 (COI) barcode of animals [10].

## Conclusions

Given the poor accessibility of high-throughput sequencing data and environmental metadata, there is an urgent need for a centralized system of standardized data deposition and management, an issue that affects many areas of biodiversity research. For example, the Global Soil Biodiversity Initiative (GSBI) has taken steps to standardize and integrate sequence information with classical morphology data [1]. Although there has been no clear leadership, these efforts demonstrate that the research community favours the implementation of standards and is prepared to move towards global guidelines. Regarding sequence data, we argue that these should be made available both in raw and quality-filtered formats that also allow easy access for non-bioinformaticians. Communication of taxonomic identification between studies would be enabled with a permanent DOI-based naming system of OTUs [9, 10]. Standardization of sequence data, metadata and taxonomic communication will greatly improve our understanding of global biodiversity and autecology of species.

## Abbreviations

BOLD Systems: Barcode of Life Data Systems
DOI: digital object identifiers
HTS: high-throughput sequencing
INSDC: International Nucleotide Sequence Database Collaboration
ITS: internal transcribed spacer
MIMARKS: ‘Minimum information about a marker gene sequence’
OTUs: Operational Taxonomic Units
QIIME: Quantitative Insights into Microbial Ecology
